# Pharmacokinetics of cannabidiol following single oral and oral transmucosal administration in dogs

**DOI:** 10.3389/fvets.2022.1104152

**Published:** 2023-01-06

**Authors:** Giorgia della Rocca, Fabiola Paoletti, Maria Beatrice Conti, Roberta Galarini, Elisabetta Chiaradia, Monica Sforna, Cecilia Dall'Aglio, Angela Polisca, Alessandra Di Salvo

**Affiliations:** ^1^Department of Veterinary Medicine, University of Perugia, Perugia, Italy; ^2^Centro di Ricerca sul Dolore Animale (CeRiDA) - Research Center on Animal Pain, Department of Veterinary Medicine, University of Perugia, Perugia, Italy; ^3^Istituto Zooprofilattico Sperimentale dell'Umbria e delle Marche, Perugia, Italy

**Keywords:** cannabidiol, CBD, dog, oral administration, oral transmucosal administration, OTM, pharmacokinetics

## Abstract

**Introduction:**

In the last few years, different formulations containing cannabidiol (CBD) were tested with regard to its efficacy on chronic pain, refractory epilepsy, anxiety, aggressive behavior and atopic dermatitis in dogs. CBD is generally administered orally, but its low bioavailability, probably due to a first-pass metabolism, represents a great limitation. The aim of this study was to evaluate if CBD bioavailability increases after oral transmucosal administration (OTM) compared to oral treatment.

**Methods:**

Twelve dogs diagnosed with mild chronic pain were enrolled in the study and treated once orally or OTM (6 dogs/group) with a pure CBD in oil formulation at a dosing rate of 1 mg/kg b.w. At prefixed time points, blood samples were collected to define CBD plasma concentrations vs. time profiles, and the main pharmacokinetics parameters were obtained by non-compartmental model.

**Results:**

CBD Cmax, Tmax, terminal half-life and AUC_0 − t_ were 206.77 ± 167 and 200.33 ± 158.33 ng/mL, 2.17 ± 0.98 and 1.92 ± 1.11 h, 2.67 ± 0.53 and 2.62 ± 0.64 h, 647.51 ± 453.17, and 536.05 ± 370.21 h^*^ng/mL, following oral and OTM administration, respectively. No significant difference in pharmacokinetic parameters were observed between treatments.

**Discussion:**

The OTM administration did not increase cannabidiol bioavailability compared to oral treatment. The almost perfectly superimposable mean plasma concentrations of cannabidiol following the two treatments suggests that CBD is not able to be adsorbed by the oral mucosa or that its absorption is very scarce, and that CBD is swallowed and absorbed in the gastrointestinal tract.

## Introduction

In the recent years, a growing interest has raised toward the use of Cannabis sativa extracts in veterinary medicine for the treatment of several type of pain, refractory epilepsy, anxiety, aggressive behavior or atopic dermatitis (1, 2). An online anonymous survey conducted by Kogan et al. (3) outlined that several pet owners were inclined to administer cannabis products to their animals due to the feeling that the efficacy was comparable to that obtained with conventional drugs. For this reason and due to the involvement of the endocannabinoid system in the pain pathways, several clinical studies investigated the efficacy of cannabis derivatives, in particular cannabidiol (CBD), on osteoarthritic chronic pain in dog. The oral administration of different CBD oils (as a sole treatment or as add-on to other analgesic drugs), generally at doses ranging between 1 and 2 mg/kg every 12 h for at least 4 weeks, resulted in a significant reduction of pain scores, an improvement of mobility and of quality of life as well as a decrease of inflammatory serum biomarkers (4–7).

Cannabidiol is generally administered orally, but its low bioavailability, lesser than 19% in dog (8), is a great limitation. One factor that influences the CBD concentration in the systemic circulation following oral administration is its formulation. Several studies have evaluated the pharmacokinetics of CBD after its oral administration as dry raw material in gelatin capsules, microencapsulated oil beads, soft chews, hemp extracts mixed with different oil types and CBD enriched cannabis herbal extracts. The CBD oil-based formulations and soft chews resulted in higher plasma concentrations (4, 8–12), indicating that the type of formulation largely influences the oral absorption of CBD.

Due to its lipophilic nature, CBD undergoes to extended metabolism as proved by Samara et al. (13) which identified several CBD metabolites in dog urine following its intravenous administration. The first-pass metabolism is believed to be one of the most plausible causes of the scarce oral CBD bioavailability (2).

An alternative route of administration that might improve the bioavailability of CBD bypassing the first-pass metabolism is the oral transmucosal (OTM). This route of drug's administration does not require particular restriction of the animal or specific skills of the owner when compared to parenteral administrations, it is painless and non-invasive and it is successfully applied in veterinary medicine to manage pain or sedate animals (14, 15). Indeed, it was recently used in a clinical study on efficacy of CBD in dogs affected by osteoarthritis, resulting in an improvement of pain scores and quality of life (16). The OTM administration could also minimize the great individual absorption variability usually observed following oral administration (4, 9, 10). The cause of this variability may be due to gastric pH, emptying time, differences between young and old in gastrointestinal anatomy and eventual presence of food and its composition in the gastrointestinal tract (17), all factors not influencing the OTM administration. An increase in CBD blood concentrations following OTM route could also allow the reduction of the administered dose with consequent containment of the cost of therapy.

The aim of this study was to evaluate the pharmacokinetics of a CBD oil-based formulation following single oral and oral transmucosal administration in the canine species, hypothesizing that CBD bioavailability was increased after OTM with respect to oral treatment.

## Materials and methods

### Animals and treatment

Twelve dogs (4 females and 8 males) of various breeds, weighing 24.4 ± 9.4 kg (mean ± standard deviation), and of 8.4 ± 4.7 years of age, were enrolled in the study (Table 1). The animals were referred to the Veterinary Teaching Hospital of the University of Perugia (Italy) and diagnosed by the veterinary clinician with mild chronic pain due to osteoarthritis (10 subjects) and Inflammatory Bowel Disease (2 subjects); no other concomitant pathologies were detected by physical exam; the hematological and biochemical parameters related to the liver and kidney functions were in the normal range. At the time of enrollment, dogs were not receiving any pharmacological treatment.

**Table 1:** Age, weight, sex, breed, disease of recruited animals, and pharmacological treatments.

**Age** ** (years)**	**Weight** ** (kg)**	**Sex**	**Breed**	**Disease**	**Drops administered**	**Dose (mg/kg)**
**Oral**						
9	36.5	F	Labrador	OA	10	0.93
2.5	36	M	Mixed	OA	10	0.94
11	22	M	Mixed	OA	7	1.08
13	12.3	M	Mixed	OA	4	1.10
1.5	20.7	M	Mixed	OA	6	0.98
8	23.3	M	Mixed	IBD	7	1.02
**OTM**						
4	17.9	M	Breton	OA	5	0.95
12	19.2	F	Border collie	OA	6	1.06
4	36	M	Dobermann	IBD	11	1.04
17	12.4	M	Mixed	OA	4	1.09
10	20	F	Border collie	OA	6	1.02
9	36.6	F	Labrador	OA	10	0.93

OA, osteoarthritis; IBD, Inflammatory Bowel Disease.

All dog's owners were interested to administer CBD as an alternative to traditional treatments and gave their written consent to participate to the study, previously approved by the Bioethical Committee of the University of Perugia (on 2nd September 2019 with protocol number: 2019 14/R).

Before the CBD treatment, food and water were withdrawn for 12 and 2 h, respectively, and an IV catheter was aseptically inserted into the right cephalic vein. This was considered as the most appropriate site for collecting blood sample after OTM administration, as the jugular vein, usually used in pharmacokinetic study, collects buccal veins, thus overestimating drugs' plasma concentrations (18, 19).

A 10% CBD oil-based formulation was prepared by an authorized pharmacy using synthetic CBD crystals of pharmaceutical grade (Cannabidiol Pharma, purity grade 100.7%; Metapharmaceutical Industrial SL, Barcelona, Spain) in medium-chain triglycerides (MCT) oil. Dogs were randomly assigned to the oral or OTM treatment group (6 dogs/group) and administered with 1 mg/kg b.w. of CBD. The 10% CBD oil allowed to administer a limited number of drops (range: 4–11 drops) to all animals, favoring an appreciable dosage correctness (Table 1). When given orally, the CBD oil was added to a small amount of commercial dry food, while for the OTM administration the CBD oil was instilled along the lateral gingiva and a gentle massage was applied to the dog's cheek to promote the transmucosal absorption of the drug. Two hours after treatment, dogs were allowed to eat their meal.

Before treatment and at prefixed post-administration time-points (0.25, 0.5, 1, 1.5, 2, 4, 6, 8, and 10 h) blood sample were collected from the cephalic vein in tubes containing sodium citrate as anticoagulant and centrifuged at 3,500 rpm; the obtained plasma samples were then stored at −80°C pending analytical determinations.

### Quantification of CBD in plasma

#### Chemicals and reagents

Cannabidiol (CBD, cod C-045-1ML) and its deuterated internal standard cannabinol-d3 (CBD-d3, cod C-084-1ML) were purchased from Sigma-Aldrich (St. Louis, MO, USA) as methanolic solutions at concentrations of 1,000 and 100 μg/mL, respectively. Working solutions were then prepared diluting the commercial products with MeOH.

MeOH, acetonitrile, n-hexane (all LC–MS grade) were obtained from Honeywell (Charlotte, NC, USA), while water and formic acid were purchased from VWR International (Radnor, PA, USA).

#### Analytical determination of CBD in canine plasma

CBD was extracted from canine plasma using the protocol suggested by Zgair et al. (20). Briefly, 0.3 mL of canine plasma was added into a 15 mL Falcon tube with 30 μL of a solution of CBD-d3 (0.1 μg/mL) in MeOH. The samples were subjected to protein precipitation with 1.2 mL of acetonitrile and left at −20°C for 5 min. Water (1.2 mL) was added to each sample prior to the addition of 6 mL of n-hexane performing liquid-liquid phase extraction. The n-hexane layer was collected and then evaporated at 30°C under nitrogen stream. Finally, the dry residue was resuspended with 0.3 mL of MeOH/H_2_O 80/20 (v/v) with 0.1% of formic acid and, after centrifugation, the sample was transferred into a glass vials and injected. LC-MS/MS measurements were performed by a Surveyor LC pump, coupled with a triple quadrupole mass spectrometer (TSQ Quantum Ultra, Thermo Fisher, San Jose, CA, USA), equipped with an electrospray source operating in positive ionization mode. Separation was achieved on a Kinetex C8 column (100 × 2.1 mm, 2.6 μm) which was connected to a guard column Kinetex C8 (2.1 × 3 mm), both from Phenomenex (Torrance, CA, USA). The mobile phases were water (A) and MeOH (B) both containing HCOOH 0.1%. The gradient profile was as follows: (1) 0–1 min, 60% B; (2) 1–7 min, to 80% B; (3) 7–9 min, to 100% B; (4) 9–14 min, 100% B; (5) 14–15 min, to 60% B, and (6) 15–22 min, 60% B. The total run time was 22 min. The column temperature was set at 40°C, the flow rate at 0.25 mL/min and the injection volume was 5 μL. Analytes were detected using multiple reaction monitoring (MRM) selecting the following transitions: CBD 315.2 m/z → 193.1 m/z, 315.2 m/z → 123.0 m/z and 315.2 m/z → 259.2 m/z; CBD-d3 (IS) 318.2 m/z → 196.1 m/z. In each analytical batch, eight concentration points (0, 2.5, 5, 10, 50, 75, 100, 150, and 200 ng/mL in MeOH) were injected as calibration curve. CBD was quantified applying the isotopic dilution technique.

Five replicates of canine plasma samples were analyzed at five spiking concentrations (1, 2.5, 10, 75, and 150 ng/mL) on two different days. Within-run and between-run precision were in the range 2.3–7.0% and 4.9–10.4%, respectively. Accuracy was always from 85 to 115%. The lower (LLOQ) and upper (ULOQ) limit of quantification were 1 and 150 ng/mL, respectively. Samples with concentrations higher than 150 ng/mL were afresh extracted, introducing a dilution factor of 10 fold, and reanalyzed.

### Pharmacokinetic and statistical analysis

The homogeneity of groups with respect to age and weight was verified by Kruskal–Wallis test while that with respect to sex by exact Fisher test.

The time/concentration curves obtained by each dog were analyzed by a non-compartmental model using the PK-Solver programme (21). The areas under the concentration-time curves from 0 to the last time (AUC_0 − t_) were calculated using the trapezoidal method.

The non-parametric Kruskal–Wallis test was applied to statistically compare the pharmacokinetic parameters between the two groups of treatment. All statistical analyses were conducted by Statistics for Data Analysis powered by SPSS version 25 (SPS srl, Italy). Differences were considered significant when *p* < 0.05.

## Results

**Figure 1:**
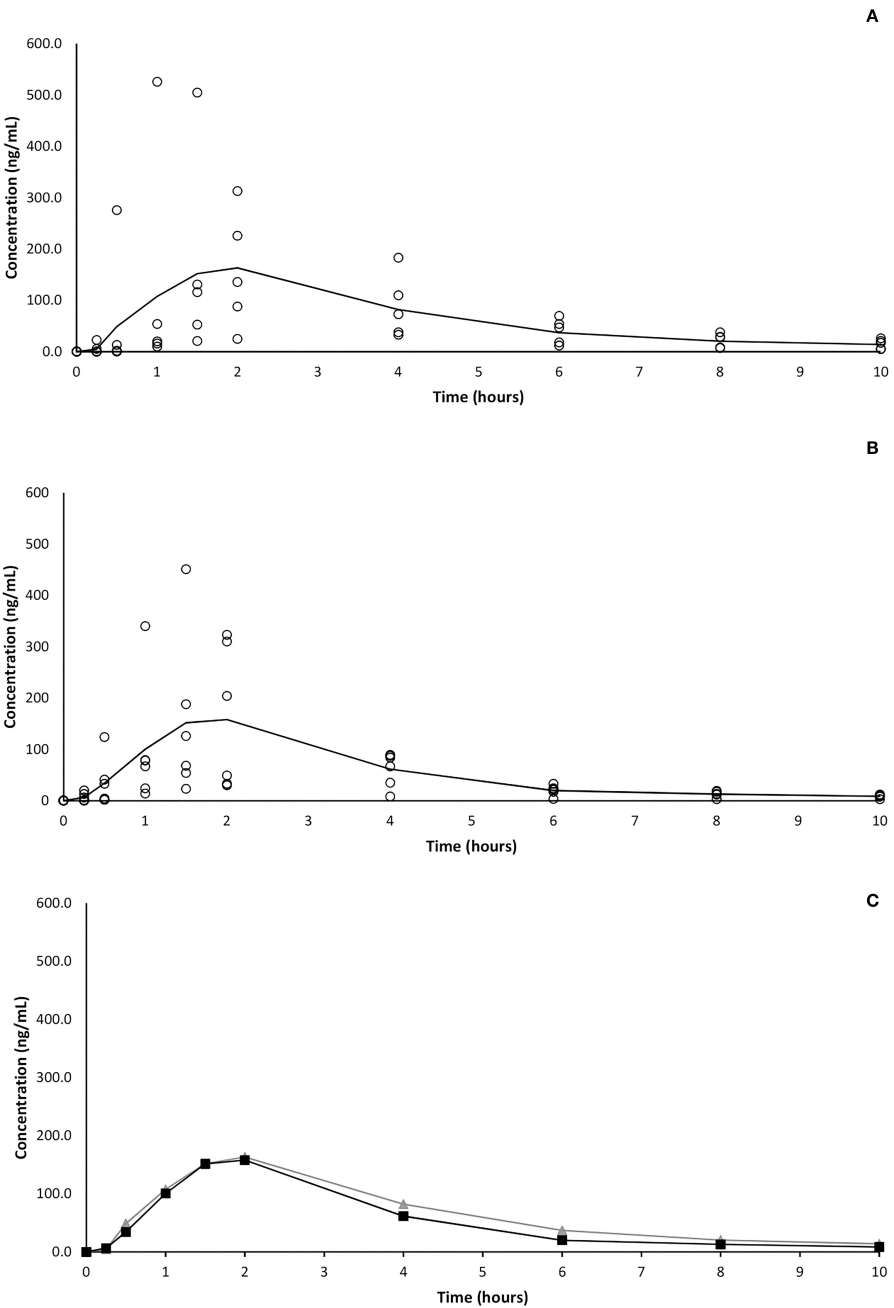
Average (solid line) and single (circles) CBD plasma concentrations vs. time following single oral **(A)** and OTM **(B)** treatment and comparison **(C)** of mean concentrations of the two different route of administration (oral in gray and OTM in black).

At the first experimental time point (15 min) CBD was detectable in plasma of 2 and 4 subjects following oral and OTM treatment, respectively. Thirty minutes after OTM administration, CBD was detected in all dogs, while only in five out of 6 subjects in the orally treated group, where CBD was detectable in all subjects 1 h post administration. The CBD plasma peak (Cmax) was achieved between 1 and 4 h (Tmax) in both treatment groups and ranged between 73 and 526 and 67 and 451 ng/mL following oral and OTM administration, respectively. At the last experimental time-point (10 h after the administration), CBD was detectable in all subjects in variable concentrations ranging from 5 to 26 and from 3 to 12 ng/mL after oral and OTM treatment, respectively. A large intersubjective variation in CBD blood concentrations was obtained at almost all the scheduled sample times as shown in Figure 1 in which CBD plasma concentrations vs. time plots of the two groups of treatment are represented.

Following non-compartmental analysis, the extrapolated percentage of the area under the curve (AUC) of one dog in the oral group was >20% (26.8%), therefore the pharmacokinetic parameters depending on terminal rate constant of this subject were considered unreliable and excluded, while parameters such as Cmax, Tmax and AUC_0 − t_ were maintained.

Table 2 shows the main pharmacokinetic parameters obtained after oral and OTM treatment with CBD. No significant differences in pharmacokinetic data resulted between the two routes of administration.

**Table 2:** Main pharmacokinetic parameters obtained following oral and OTM administration of CBD at 1 mg/kg in dogs (6 dogs/group).

**Parameter**	**λz**	**t_1/2_**	**T_max_**	**C_max_**	**AUC_0-t_**	**AUC_0−∞_**	**AUC_Estr_**	**AUMC_0−∞_**	**MRT**
**Unit**	**1/h**	**h**	**h**	**ng/mL**	h*ng/mL	h*ng/mL	**%**	h^2^*ng/mL	**h**
**Oral**									
Dog 1	0.33	2.09	2	135.60	394.33	410.57	4.12	1371.46	3.34
Dog 2	0.21	3.27	2	192.00	482.00	524.49	8.81	2177.71	4.15
Dog 3	0.22	3.17	2	88.00	251.00	273.84	9.10	1167.83	4.26
Dog 4	0.29	2.40	1	526.00	1419.00	1477.84	4.15	4431.41	3.00
Dog 5	0.25	2.80	2	226.00	971.00	1076.01	10.81	5324.44	4.95
Dog 6	n.a.	n.a.	4	73.00	367.75	n.a.	n.a.	n.a.	n.a.
**Mean**	**0.25**	**2.67** ^*^	**2.17**	**206.77**	**647.51**	**752.55**	**7.40**	**2894.57**	**3.94**
**(S.D)**	**(0.05)**	**(0.53)** ^**§**^	**(0.98)**	**(167.07)**	**(453.17)**	**(507.15)**	**(3.08)**	**(1876.27)**	**(0.78)**
**OTM**									
Dog 1	0.25	2.74	1.5	451.00	1128.50	1175.95	4.20	3501.87	2.98
Dog 2	0.21	3.27	2	323.00	783.10	825.59	5.44	2853.82	3.46
Dog 3	0.38	1.83	1	79.00	150.08	158.00	5.28	447.32	2.83
Dog 4	0.27	2.52	4	67.00	275.75	304.88	10.56	1601.08	5.25
Dog 5	0.25	2.74	2	204.00	577.00	608.63	5.48	2282.41	3.75
Dog 6	0.21	3.24	1	78.00	301.88	353.22	17.01	1840.16	5.21
**Mean**	**0.26**	**2.62** ^*^	**1.92**	**200.33**	**536.05**	**571.04**	**8.00**	**2087.78**	**3.91**
**(S.D)**	**(0.06)**	**(0.64)** ^**§**^	**(1.11)**	**(158.34)**	**(370.21)**	**(379.74)**	**(4.95)**	**(1059.59)**	**(1.07)**

AUC_0−*t*_, area under serum concentration-time curve; AUC_**0−∞**_, area under serum concentration-time curve from time 0 to infinity; AUC_extr_, area under the concentration-time curve extrapolated from t_last_ to ∞ in % of the total AUC; AUMC_**0−∞**_, area under moment curve; C_max_, maximum concentration observed; λz, terminal rate constant; MRT, mean residence time; T_max_, time of maximum concentration observed; ${\text{t}}_{\frac{1}{2}}$, terminal half-time; ^*^, Harmonic mean; ^§^, pseudo-standard deviation; n.a, not available.

## Discussion

Previous studies evaluating the pharmacokinetics of CBD in dogs employed dosages higher than those usually applied in clinical practice (9, 11, 22). From anecdotal data and published studies on the efficacy of CBD in dogs in the treatment of osteoarthritis and epilepsy, oral doses between 1 and 2 mg/kg every 12 h are generally used successfully (2). According to the aphorism “Start low, go slow, stay low” (23), a dose of 1 mg/kg of CBD was chosen by the clinician responsible for the enrollment of animals in the present study.

A 10% formulation of CBD in MTC oil was used in the present study to permit the administration of reduced volumes of solution considering the dogs' weight range, thus avoiding losses outside the mouth when given OTM (16). Moreover, besides preventing the oxidative degradation and the decrease of cannabinoid's concentration better than other oils (24), MTC oil is flavorless, limiting ptyalism and vomiting (16). When given orally, CBD oil was mixed with a small amount of dry food to facilitate the administration of the drug and as a food bolus is reputed to enhance the gastrointestinal absorption of very lipophilic substances such as CBD (2). In a human study, the administration of CBD with a high-fat meal, resulted in Cmax and AUC over 4 times greater than in fasted condition (25). It is believed that food enhances the absorption of lipophilic drugs by increasing their permanence in the gastrointestinal tract, their solubilization and their lymphatic transport by lymph lipoproteins (26). Deabold et al. (10), suggested that the same phenomena might incur in dogs, where the administration of CBD formulated as soft chew, considered a food matrix, resulted in Cmax and AUC about 3 times greater than that observed in a previous published study performed with CBD oil (4). On the other hand, a study where the pharmacokinetic of Bedrocan**^®^**, a cannabis oil extract, was performed in fasting and fed dogs, the latter showed a longer Tmax and a lower Cmax compared with the fasted group, and a relative oral bioavailability of THC of 48.22% (27). The Authors speculated that being THC a lipophilic compound, it should have increased bioavailability in the fed condition. However, the lipophilicity of the olive oil formulation might have increased the THC absorption in the fasting dogs, while, in fed status, THC might have been adsorbed by food showing a longer Tmax (27). This speculation could also apply to CBD. It should be highlighted that studies where the relative bioavailability of CBD orally administrated to fed and fasted dogs is compared are not available.

In the present study, after oral administration of CBD mean Cmax and AUC values were higher than those obtained in previous published pharmacokinetic studies where CBD oil was orally administered to fed or fasted dogs, when normalized for the dose (4, 9, 11, 12). This difference could be due to the great individual variability in CBD plasma concentrations observed in the present and previous studies, but also to the different administered oral formulations. Indeed, while other Authors used formulations containing also other cannabinoids, in the present study a pure CBD in MTC oil formulation was employed. In a study where CBD was orally administered both as a full-spectrum extract or as a pure molecule to mice, higher mean peak plasma (304 ± 28 vs. 60 ± 6 ng/mL) and AUC value (104 vs. 43 μg^*^min/mL) were observed following treatment with pure CBD (28). In the same study, a shorter half-life (217 vs. 484 min) after treatment with pure CBD was also observed, so the Authors speculated that the presence of other cannabinoids in the formulation might influence the rate of CBD biotransformation (28). Similarly, in the present study the terminal half-life (2.67 h) was shorter than that obtained by Gamble et al. (4) and Wakshlag et al. (12) (more than 4 h) when a CBD:CBDA (1:1)- predominant hemp oil was used. On the other hand, it was similar to that obtained following oral administration of a CBD infused oil (9) or CBD enriched cannabis herbal extract (11). As the concentration of CBDA in these last two formulations was not declared, it is possible to hypothesize that the presence of CBDA may be responsible for a slower clearance of CBD. It is important to underline that the Tmax obtained in the present study was quite close to that observed in the above cited studies (4, 11, 12) and consequently that the differences in terminal half-life values are not due to different absorption rates due to the formulation' differences.

Even if it is rather complicated to compare the pharmacokinetic of CBD following oral administration from different studies, because of several factors that might influence the plasma concentrations of the drug, it is generally believed that CBD has a low oral bioavailability due to a first-pass metabolism and a scarce absorption (29). Furthermore, also an absorption rate slower than the elimination rate could be as responsible for the reduced CBD plasma concentrations (22, 24). More studies exploring the influence of formulation (i.e., pure CBD, hemp extract or CBD enriched hemp extracts) and “food effect” on oral pharmacokinetics of CBD in dogs are warranted.

To the authors' knowledge, this is the first pharmacokinetic study comparing OTM vs. oral administration of CBD in dogs. We hypothesized that CBD bioavailability could increase after OTM with respect to oral treatment. Indeed, the drug absorption by the OTM route should allow its rapid uptake across the oral mucosa and avoid its first-pass metabolism and any other problems related to its absorption in the gastrointestinal tract, consequently increasing its bioavailability and allowing to a dose reduction (30, 31).

Contrarily to our hypothesis, the OTM administration of CBD did not improve its bioavailability. The possibility that salivation and subsequent swallowing could have affected the drug's transmucosal absorption cannot be ruled out (30). However, in this case a secondary drug plasma peak should have been observed, while in the present study no double peaks resulted following OTM administration. Even if we cannot exclude to have missed the sample time point of the secondary peak, the almost perfectly superimposable mean plasma concentrations of CBD following OTM and oral administration (as shown in Figure 1C), suggests the inability, or a reduced ability, of CBD to be absorbed through the oral mucosa and that probably it was swallowed and absorbed at the gastrointestinal tract level. Comparing some pharmacokinetic studies on CBD following its administration as an oral mucosal spray in fed and fasted humans, Itin et al. (32) supposed the presence of a “food effect.” However, the presence of food in gastrointestinal tract should not influence the plasma profile of a drug following its transmucosal application, letting these Authors to hypothesize that the majority of CBD was swallowed instead of passing through the oral mucosa. A possible explanation of the low OTM absorption of CBD could lie in the fact that while a good candidate for OTM delivery should have a log P above 2.0, a higher lipophilicity, as that of CBD, which has a Log P of 5.91, could be an obstacle to its diffusion in the cell cytoplasm (30, 33).

The hypothesis of a lacked or reduced absorption of CBD through the canine mucosa is reinforced by the results obtained by Polidoro et al. (22) who administered CBD by intranasal (IN), intrarectal and oral route in dogs. As the IN and rectal route are alternative administration routes able to avoid or partial avoid the first-pass metabolism in the liver, an increase of CBD plasma concentrations was expected compared to oral administration. However, following rectal treatment, CBD plasma concentrations were not quantifiable and no significant differences between oral and IN administration were observed regarding plasma peaks and AUCs (when normalized for the dose) (22). The Authors concluded that even if the eventual presence of sneeze, nasal congestion and mucous could have reduced the absorption of CBD, it was possible that CBD was largely swallowed (22).

A limitation of the present study is that it did not detect the metabolite (7-COOH derivative of CBD) that is known to be produced in dogs (12). The quantification of CBD metabolites in canine blood after OTM concentration could be important in order to better understand the pharmacokinetics of CBD and fully attribute the results of future pharmacodynamic studies.

## Conclusions

Due to its multiple biological effects, various health benefits and lack of psychoactive properties, CBD is becoming of great interest in veterinary medicine. To better take advantage of the therapeutic effects of CBD it is important to assure that the necessary plasma concentrations to obtain therapeutic effects are achieved.

Contrarily to our expectations, the OTM administration of a pure CBD oil did not increase its bioavailability compared to oral administration. The development of innovative formulations able to enhance a fast penetration of CBD in the systemic circulation through the oral mucosa is therefore desirable.

## Data availability statement

The raw data supporting the conclusions of this article will be made available by the authors, without undue reservation.

## Ethics statement

The animal study was reviewed and approved by BioEthical Committee of the University of Perugia. Written informed consent was obtained from the owners for the participation of their animals in this study.

## Author contributions

GR contributed in the study design, sample collection, and revising the final manuscript. FP and RG performed the method validation and quantification of CBD in plasma samples. MC participated in the conceptualization of the study, coordinated the clinical phase, and revised the manuscript. EC, MS, CD, and AP participated in the study design and revised the manuscript for intellectual content. AD contributed in the study design, performed the analysis and interpretation of data, drafted the manuscript, and supervised the whole work. All authors read and approved the final manuscript.
